# Outcome of basal cell carcinoma excision with 2 mm surgical margin in Japanese patients: A retrospective study of one-step surgery

**DOI:** 10.1016/j.jpra.2024.11.010

**Published:** 2024-11-26

**Authors:** Kaori Kyono, Yoshinori Tamada, Michito Ara, Shin-Ichiro Yamagishi, Ayako Higuchi, Keiichiro Iida, Naoko Wada, Makoto Mikami, Satoshi Urushidate

**Affiliations:** a Department of Plastic and Reconstructive Surgery, Hirosaki University Graduate School of Medicine, 5 Zaifu-cho, Hirosaki City, Aomori, 036-8562, Japan; b Department of Medical Data Intelligence, Research Center for Health-Medical Data Science, Hirosaki University Graduate School of Medicine, 5 Zaifu-cho, Hirosaki City, Aomori, 036-8562, Japan; c Department of Plastic Surgery, Aomori Rosai Hospital, 1 Minamigaoka, Shirogane, Hachinohe City, Aomori, 031-8551, Japan; d Department of Diagnostic Pathology, Aomori Rosai Hospital, 1 Minamigaoka, Shirogane, Hachinohe City, Aomori, 031-8551, Japan

**Keywords:** Basal cell carcinoma (BCC), Narrow surgical margin, Risk factors, Local recurrence, One-step surgery

## Abstract

**Background:**

Basal cell carcinoma is the most common skin malignancy. The standard treatment is surgical excision with predetermined margins. Some argue that the currently recommended surgical margins are excessive, and it is questionable whether such wide surgical margins should be applied to all lesions. We statistically investigated excisions with narrow margins and tried to identify the risk factors for recurrence after one-step surgery.

**Methods:**

Basal cell carcinomas were excised at a single institute in Japan over a six-year period and the recurrence rates were retrospectively analyzed using pathological reports and case notes. We reviewed the microscopic findings of the excised specimens and examined the excisional margin status, tumor subtype, and perineural invasion.

**Results:**

Forty-eight basal cell carcinomas (45 primary and 3 recurrent) that were identified in Japanese patients were included in this study. Among the primary lesions, well-pigmented and well-defined lesions did not show any involvement of the surgical margins, perineural invasion, or development of local recurrence. Recurrent lesions were significantly associated with positive surgical margins (side margin, P<0.01; deep margin, P<0.01) during the primary operation; however, no association was found with local recurrence after re-resection. Significant differences were observed in perineural invasion and the tumor subtype, especially in the aggressive subtype (P<0.05).

**Conclusions:**

A 2 mm margin allows for the safe excision of primary lesions with well-pigmented and well-defined basal cell carcinoma in Japan. Recurrent lesions can be treated with narrow margins by reconstruction after confirmation of a negative margin, instead of performing a common resection with wide margins.

## Introduction

Basal cell carcinoma (BCC) is a common skin cancer in some countries.^1^ Metastatic BCC is extremely uncommon,^2^, ^3^, ^4^ usually slow-growing, and rarely life threatening. Because most BCCs are found in the head and neck region, careful treatment is required when skin defects are large after excision. Local infiltrations can cause severe disfigurement and, in rare cases, death.

According to the National Comprehensive Cancer Network (NCCN) guidelines, BCCs are classified into low- and high-risk groups based on the lesion site, size, pathological subtype, and previous resection as risk factors for recurrence. Wide surgical margins (>4 mm) have been recommended by the NCCN guidelines.^5^ However, some studies have reported that narrow (2-3 mm) surgical margins are sufficient. In Japan, excision with 2-mm surgical margins achieved a complete removal rate of >95% if BCCs had pigmentation and a well-defined border.^6^ The recurrence rate of well-pigmented BCCs resected with 3 mm margins was 1% after several years of follow-up.^7^

There is a quantitative argument in the literature regarding optimal surgical margins. However, some studies did not conduct a detailed follow-up after excision.

This study aimed to assess whether 2 mm margins provide adequate tumor margin control and a satisfactory long-term recurrence rate according to the clinical and histopathological findings.

## Methods

This retrospective study was conducted at the Aomori Rosai Hospital in Hachinohe, Japan, from January 1, 2016, to May 31, 2022. A senior plastic surgeon excised 95 lesions during the study. Out of 95 lesions, 47 lesions were excluded because the duration of follow-up was less than one year. A total of 48 lesions from all Japanese patients were included in the statistical analysis. Patients were followed up for more than one year, and underwent skin examinations every 3-12 months. No Caucasians were included in this study. This single-center retrospective study followed the Strengthening the Reporting of Observational Studies in Epidemiology guidelines and was based on data obtained from the medical records.

Data on the patient characteristics included age, sex, previous history of skin disease treatment using psoralen and UVA lights (PUVA), primary or recurrent disease, status of immunosuppression by organ transplantations, and sites of prior radiotherapy. Data regarding the anatomical site, pathological characteristics, duration of follow-up, local recurrence, and operation notes were obtained from the hospital computer database system. Pathological data included tumor size, tumor thickness, surgical margins, perineural infiltration, and subtype. They were obtained from a retrospective review of the postsurgical specimens. The tumor size was calculated using the axial dimensions. Tumor pigmentation was classified as well-pigmented if there was a dark black color on more than 95% of the surface area. Clinical tumor borders were classified as well defined if the entire margins were clearly demarcated. Evaluations were performed retrospectively using photographs. Presurgical ultrasound sonography, confocal microscopy (RCM), and optical coherence tomography were not used to determine the tumor edge deep or side margins. If the tumor did not have any color or we could not predict the tumor vertical or invasion level, we used the instruments for the preoperative examination, including dermatoscopy or magnetic resonance imaging. However, cases evaluated for presurgical planning were excluded. We classified and compared the number of BCCs in the primary (well-pigmented and well-defined, well-pigmented and poorly defined, poorly pigmented and well-defined, poorly pigmented and poorly defined) and recurrent (well-pigmented and well-defined, poorly pigmented and well-defined) groups.

All specimens were fixed in 10% buffered formalin after excision and then were examined using routine hematoxylin and eosin (HE) staining.

The pathological subtype pattern was classified as nonaggressive (superficial, nodular, and Pinkus types) or aggressive (micronodular, infiltrating, and morpheic types) based on the WHO classification.^8^ The morpheic type is rare and is included in the aggressive subtype. Horizontal surgical margin assessment was performed using 2-mm-thick or more thinly serial sections perpendicular to the horizontal surgical margins. Recurrent lesions were selected for thinning. Vertical surgical margins were assessed in all of the sections. We conducted a statistical analysis to examine the association between size and the pathological findings, to estimate the likelihood of positive surgical margins and the local recurrence, and then to compare the rates of differences with positive surgical margins across different tumor subtypes.

Standard surgical excision was performed with 2-mm narrow side margin.

Deep excisional margins included subcutaneous fat, fascia, or sometimes the muscle that was deeper than the deepest edge of the tumor. If the tumor was localized in the orbital region, then it was mostly determined at a deep level up to the muscle. In particular, recurrent lesions and the side and deep margins were evaluated as thinly as possible to make detailed histologic visualized assessments of alternative Mohs surgery. The follow-up was continuous and diligent. Postoperative radiotherapy or an additional excision is performed in patients with positive margins. The risk of local recurrence and necessity for re-excision were explained. After a full discussion, some patients selected conservative treatment, such as observation with a wait-and-see approach, with surgical treatment considered when local recurrence was identified.

All analyses were conducted using nonparametric statistical tests.

Seven nominal variables were included: side margin, deep margin, perineural invasion, border status, presence of pigmentation, histological subtype (aggressive or nonaggressive), and previous BCC occurrence. Fisher's exact test was performed for nominal variables, and a multiple comparison test with Benjamini–Hochberg correction was used for groups with more than three histological subtypes. Differences between tumor size, side and deep margins, and perineural invasion, and the development of local recurrence were analyzed using the Mann–Whitney test. All data were analyzed using the R 4.3.1 software program (CRAN, freeware). Statistical significance was set at P <0.05.

## Results

Forty-four Japanese patients were included in this study. The mean patient age was 71 years (range: 42-90 years), calculated based on the age at the time of surgery. There was a male predominance, with 23 males (52.2%) and 21 females (47.7%) included in the cohort. No medical history of organ transplantation, long-term PUVA therapy, or radiation therapy was reported. If the patients had other sites of BCCs, each lesion was counted separately.

A total of 45 primary and 3 recurrent BCCs were excised and histologically evaluated. Table 1 describes the histological distribution, and Table 2 shows the patient demographics with positive margins. Three patients had two or more BCCs. One patient had two primary BCCs, while another developed two recurrent lesions at different times (Tables 2, patients’ numbers 6 and 7). The third patient had two primary BCCs, one of which recurred (Tables 2, patient numbers 1 and 8).

**Table 1:** Pathological Findings According to Presence of pigmentation, and the Tumor Border.

	Positive side margin	Positive deep margin	Perineural invasion	Local recurrence
Primary BCC (total 45 lesions)				
Well pigmented and well-defined (23)	0	0	0	0
Well pigmented and poorly defined (3)	0	0	1	0
Poorly pigmented and well-defined (10)	0	0	0	0
Poorly pigmented and poorly defined (9)	1	1	2	1
Recurrent BCC (total 3 lesions)				
Well pigmented and well-defined (2)	2	1	0	1
Poorly pigmented and well-defined (1)	1	1	1	0

BCC, basal cell carcinoma.

**Table 2:** Patient demographics who have a positive surgical margin and perineural invasion.

Patient no.	Pigmentation	Clinical tumor border	Primary or recurrent	Histological subtype	Positive margin area	Perineural invasion	Additional treatment	Total follow-up time (months)
1	Poorly	Poorly	Primary	Mixed (nodular and Infiltrating)	−	+	−	52
2	Poorly	Poorly	Primary	Nodular	side	−	−	47
3	Poorly	Poorly	Primary	Nodular	Deep	−	−	47
4	Poorly	Poorly	Primary	Nodular	−	+	−	21
5	Well	Poorly	Primary	Mixed (nodular and micronodular)	−	+	−	17
6	Well	Well	Recurrent	Nodular	Side	−	−	55
7	Well	Well	Recurrent	Micronodular	Both	−	Radiation	34
8	Poorly	Well	Recurrent	Mixed (nodular and infiltrating)	Both	+	Radiation	26

−, negative; +, positive; Patient numbers 1 and 8, 6 and 7 are all the same patient.

Of the primary BCCs, 23 lesions were well-pigmented and well-defined, with a mean tumor size of 8.7 mm (median size: 9.8 mm). None of these 23 lesions showed a positive surgical margin, perineural invasion, or the development of local recurrence within a follow-up period of 36 months. Three lesions were well pigmented and poorly defined, and perineural invasion was identified in one of the three cases. Ten lesions were poorly pigmented but well defined; none of the lesions showed positive surgical margins. Nine lesions were poorly pigmented and poorly defined. Two of these lesions had positive excisional margins, one had positive side margins, and one had positive deep margins. Microscopically, these surgical margins were very close to the excisional margin, but the tumor foci were not divided or cut. Therefore, we conducted a thorough follow-up instead of any additional excision. These two margin-positive sites showed no signs of local recurrence within 47 months of follow-up.

Table 3 shows the total number of lesions at each lesion site. The nasal area was the most frequently affected by both primary and recurrent BCCs. The ala is a particularly common region of the nose. Among the non-nasal areas, the cheeks were the most common site.

**Table 3:** Total Number of Tumor Sites.

Tumor site	Tumor no.	Tumor site	Tumor no.
Head and neck region	40	Trunk and extremities	8
Nose	14	Forearm	2
Ala (10)		Back	2
Dorsum (3)		Chest	1
Tip (1)		Abdomen	1
Cheek	6	Armpit	1
Others	20	Upper arm	1

**Figure 1:**
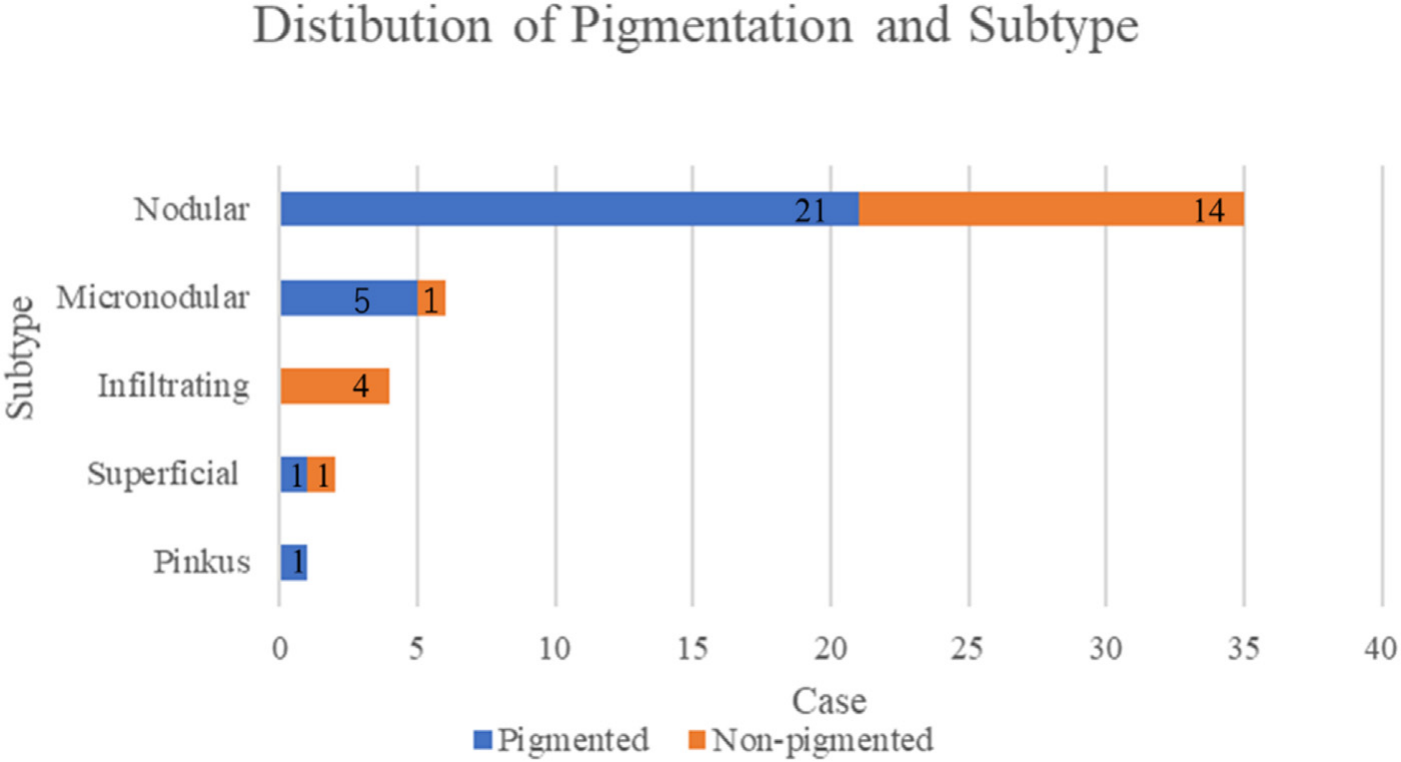
Distribution of pigmentation according to histological subtype. More than half of the nodular lesions were well-pigmented. All the infiltrating types showed poor pigmentation.

**Figure 2:**
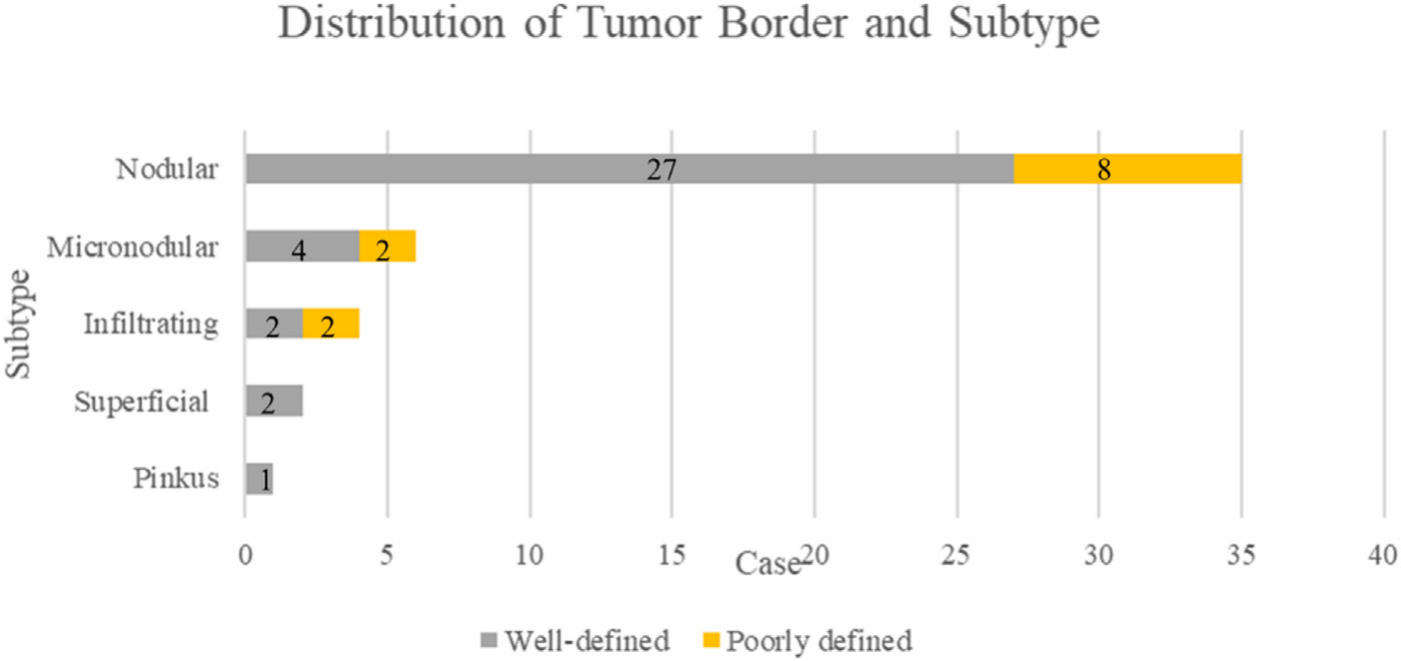
Distribution of the tumor border status according to the histological subtype. Most of nodular type cases were well-defined. The micronodular and infiltrating type included more poorly pigmented cases than the nodular type.

Clinically, 58.3% of BCCs were well-pigmented and 75% were well-defined. Figure 1, Figure 2 show the distributions of pigmentation and the border status according to the subtype. These figures show that most of the nodular types were well-pigmented (n=21), well-defined (n=27), and well-demarcated.

Of the three recurrent BCCs (irrespective of subtype, pigmentation, and tumor border status), all lesions were side-margin positive. Two of the three recurrent lesions showed positive deep margins. One of the three recurrent lesions demonstrated local recurrence after 19 months of follow-up.

**Figure 3:**
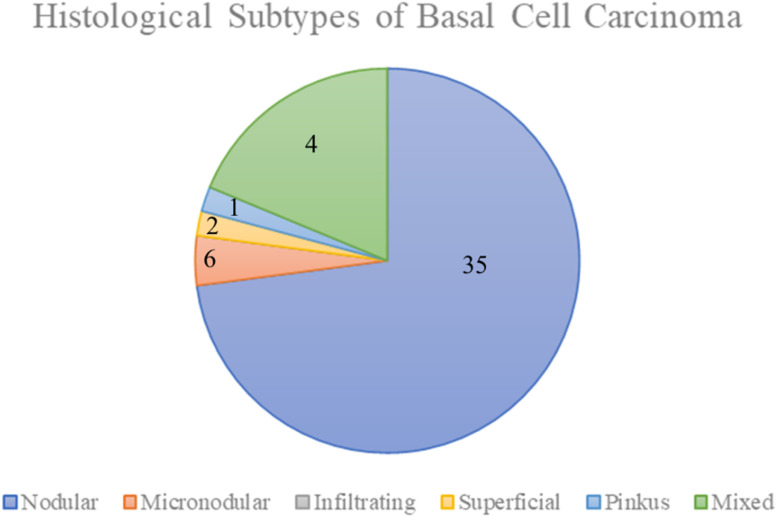
There were no simple infiltrating types because most of the infiltrating type tumors included some nodular type components. The mixed type contained some micronodular type, infiltrating type, nodular type, and superficial type components. The Pinkus type was equal to the fibroepithelial type.

**Figure 4:**
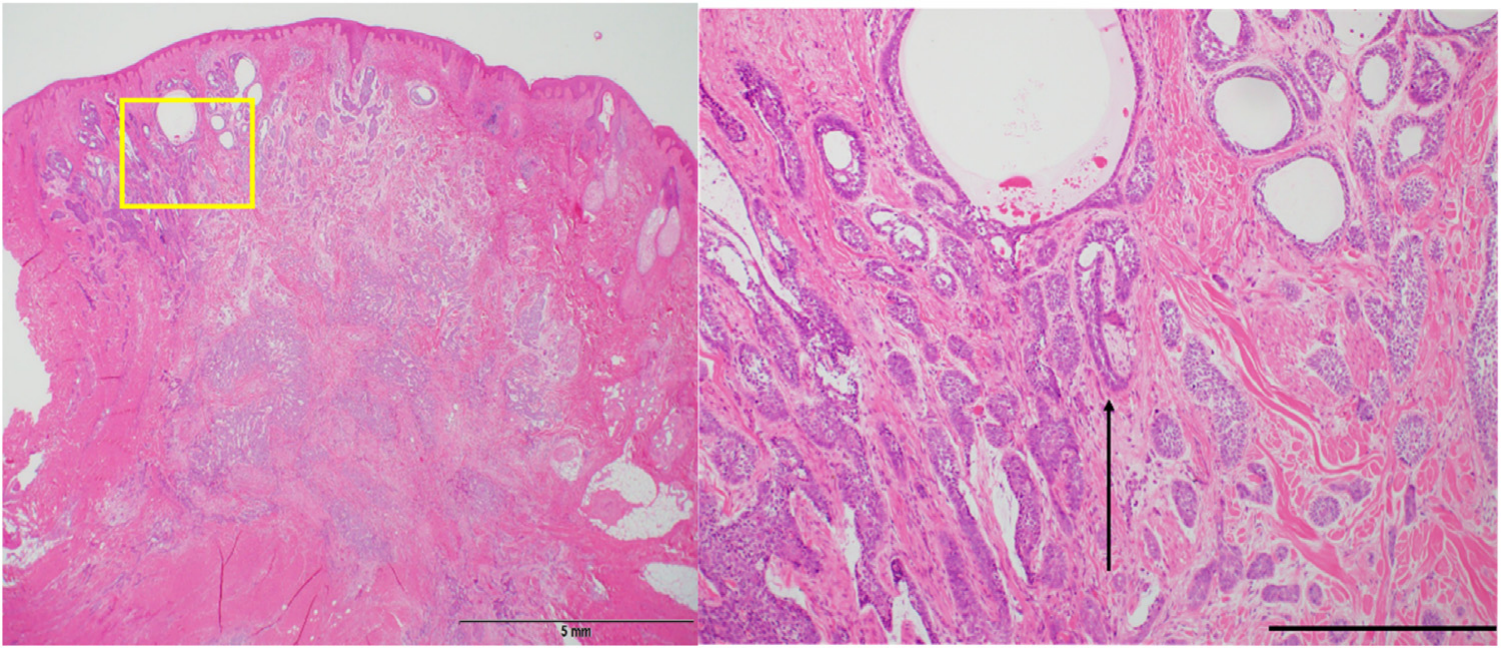
A loupe view (left). A representative microsection (yellow box, left) shows perineural invasion (black arrow, right). Scale bar=100 µm (hematoxylin and eosin staining).

As shown in Figure 3, the majority of the lesions were nodular. The mixed type is micronodular and infiltrative, with a tendency to grow invasively and become unclear. In recurrent BCCs, collagen fibers grow thickly in the stroma, and BCC cells infiltrate and destroy these stromal structures. Small BCC cells consisted of small nests that penetrated and invaded the intact tissues (Figure 4).

In a statistical analysis comparing recurrent and primary lesions, all recurrent lesions had an incomplete side excisional margin (*P*=0.0002), and two of the three lesions had an involved deep margin (*P*=0.0078). However, recurrent lesions were not significantly associated with the risk of local recurrence. There were no significant differences in margin positivity and local recurrence development between pigmented and nonpigmented lesions or between well-defined and poorly defined borders (Table 4).

**Table 4:** Estimated Positive Surgical Margins and the Development of Local Recurrence.

	Involvement of surgical margin				Development of local recurrence	
	Side margin		Deep margin			
	Tumor no. (%)	*P*	Tumor no. (%)	*P*	Tumor no. (%)	*P*
Excision of BCC previously						
No (primary)	1 (25)		1 (33)		1 (50)	
Yes (recurrent)	3 (75)	0.0002*	2 (66)	0.0078*	1 (50)	0.1223
Presence of pigmentation						
Pigmented	2 (50)		1 (33)		1 (50)	
Nonpigmented	2 (50)	1	2 (66)	0.5629	1 (50)	1
Tumor borders						
Well-defined	3 (75)		2 (66)		1 (50)	
Poorly defined	1 (25)	1	1 (33)	1	1 (50)	1
Perineural invasion						
Positive	1 (25)	0.3	1 (33)	0.2343	1 (50)	0.1613
Negative	3 (75)		2 (66)		1 (50)	

BCC, basal cell carcinoma.

Fisher's exact test was used to compare the surgical margins, perineural invasion, and local recurrence according to the tumor characteristics.

⁎ Statistically significant.

The mean size of the tumors with inadequate margins or tumors in which local recurrence was identified during the follow-up was 11 ± 4 mm, which was larger than that of tumors with complete excisional margins and those without local recurrence (mean: 8.9 ± 6.2 mm). However, there was no significant association between tumor size and either margin positivity or perineural invasion (Table 5). In addition, we analyzed the association between the tumor size and local recurrence. No significant association was observed (*P*=0.4716). No significant differences were observed between the groups.

**Table 5:** Correlation between the Pathological Findings and Tumor Size.

Pathological findings	Tumor diameter
	*P*
Side margin positive	0.3703
Deep margin positive	0.5795
Perineural invasion	0.8519

Mann–Whitney test was used to compare the tumor size.

Four cases of perineural invasion were identified in this study. In the subtype group analysis, there were no statistically significant differences in the margins between subtypes. However, the difference in perineural invasion between the aggressive and nonaggressive subtypes was statistically significant (Table 6).

**Table 6:** Summary of the Statistical Analysis Outcome.

	Aggressive type no.	Nonaggressive type no.	*P*
Side margin positive	2	2	0.1871
Deep margin positive	2	1	0.1058
Perineural invasion	3	1	0.02451*

Fisher's exact test was used to compare the surgical margins and perineural invasion between the BCC subtypes. A multiple comparison test with Benjamini–Hochberg correction was used for groups with more than three histological subtypes. The aggressive type included infiltrating and micronodular types. The nonaggressive types included nodular, Pinkus, and superficial types.

⁎ Statistically significant.

One-step surgery (e.g., primary closure, local flap, skin grafting, and secondary healing) has been used for the treatment of BCCs. The average follow-up time was 42 months, and two cases of local recurrence were observed at 12-36 months during follow-up. Two patients developed local recurrence, and additional treatments were administered to reduce the risk of tumor progression. Because these patients had a more extensive disease, radiotherapy was selected to reduce their physical burden. No local recurrence of skin lesions was observed.

## Discussion

Various methods have been used for the treatment of BCCs, such as curettage, excision with predetermined margins, excisions in combination with intraoperative rapid diagnosis by frozen section, and Mohs’ micrographic surgery (MMS).^9^, ^10^, ^11^ MMS has shown a higher cure rate for several cutaneous malignancies, and it is widely used in Europe and the United States.^9^ MMS is a surgical method that is performed worldwide.^10^

However, in Japan, surgeons performing MMS are a minority because it requires several specialized techniques, such as cutting and slicing the specimen and determining the margin status in a frozen section.^11^ Alternatively, surgical excision can be performed with predetermined margins. In Japan, one-step surgery is widely accepted.^9^^,^^12^ However, if the histological findings indicate the possibility of residual carcinoma cells or positive margins after excisional surgery, an additional excision is needed.

Some studies have recommended two-step surgery to ensure complete excision.

This study revealed that well-pigmented and well-defined primary BCCs were excised with a 2 mm margin, and a local recurrence rate of 0% in Japanese patients. While BCCs may have well-defined borders, the finding of our study suggest that they may often exhibit a greater degree of histopathological spread than of clinical tumor spread, especially in the case of recurrent lesions. When adjusting for individual cases and performing resections of high-risk BCC with a 2-mm narrow margin, either combination treatment with an intraoperative rapid diagnosis or two-step surgery with reconstruction after confirming negative surgical margins should be considered.

According to some Asian reports, the majority of resected BCCs tend to be well-demarcated (52.4%-90%), and surgical excision with a margin of <3 mm may be acceptable for Asians.^6^^,^^7^ Gulleth et al. suggested that a 3-mm surgical margin can be safely used for nonmorpheaform type (classified into aggressive subtype), tumors of <20 mm in size.^13^

Furthermore, there have been some studies on the utility of surgical margins of 1-3 mm in Japanese cohorts. Ito et al. reported side margins only in the nodular and superficial types. According to their findings, the incomplete excision rates with 2- and 3-mm surgical margins were 4.7 % and 0 %, respectively.^6^ Nakamura et al. reported that excision of high-risk BCCs with 2- and 3-mm surgical margins was associated with positive margin rates of 12% and 3.7%, respectively.^14^ Ito et al. did not discuss any other subtypes, and Nakamura et al. did not investigate the long-term prognosis. They discussed only limited specific subtypes and the postoperative follow-up. In contrast, the present study assessed six subtypes (contained mixed), and not only the results of the pathological findings, but also the long-term prognosis. Well-pigmented and well-defined primary BCCs were excised completely with a 2 mm surgical margin, regardless of subtype, and no local recurrence was identified in Japanese patients. Excisions with a 2 mm margin are widely accepted in Japan and could therefore be a safe surgical treatment for primary well-demarcated lesion.

Globally, Yusuf et al. reported a meta-analysis including BCCs in Caucasians and suggested that smaller margins were associated with a greater risk than larger margins, even if the difference was 1 mm. The relative risk of recurrence with a 2 mm margin was approximately 10 times that with a 5 mm margin, and there was a statistically significant difference. However, the mean recurrence rate is <5%.^13^ Recurrent BCCs could not be completely and safely excised with a 2 mm margin, and there was the possibility of a positive margin with the remaining tumor cells. Therefore, standard surgical excision with wide margins is required to reduce the risk of recurrence.

The nodular and superficial types sometimes contain melanin, which is well circumscribed and pigmented. However, Nakamura et al. reported that a statistically significant difference in the estimated side-margin positivity rate was observed in tumor borders excised with 2 and 3 mm margins.^15^ In this study, there were no statistically significant differences in tumor pigmentation, border, subtypes, or local recurrence.

Aggressive subtypes were more difficult to excise completely, but there was no significant difference between the pathological subtypes (aggressive or nonaggressive) in cases with positive surgical margins. Perineural invasion is associated with the risk of metastasis and local recurrence.^3^^,^^4^ There was a significant difference in the rate of perineural invasion between the aggressive and nonaggressive types, but there was no statistically significant difference in the rate of local recurrence.

This study revealed a statistically significant difference between recurrent BCCs and positive surgical margins at primary surgery, but not in the rate of local recurrence after re-resection. Linus et al. reported that one of the strongest risk factors for recurrence was a lesion that recurred after a previous excision.^16^ Matsushita et al. reported that the histological pattern and tumor size were significantly correlated with a positive vertical edge.^17^ Masud et al. found that the residual tumor rate (62.9%) was comparatively higher than the recurrence rate (30%-40%). They suggested that not all residual tumors remaining after primary excision develop into a recurrence of BCC.^18^ In this study, recurrent BCC was the only significant risk factor associated with the involvement of surgical margins.

Several strategies for the treatment of positive margins have been documented in the NCCN guidelines, and re-excision is recommended immediately, if clinically feasible.

Matthias et al. reported that recurrent sites reconstructed with primary closure, STFGs, and FTSGs, were diagnosed at 10.5 ± 5.5 months after surgery. BCC recurrence was identified at sites treated with local flaps after an average of 32.3 ± 4.7 months. These differences were statistically significant. The risk of recurrence increased significantly as the number of surgeries required to complete pathological resections increased.^19^

Surgery for recurrent BCCs has been suggested to involve excision with a wide surgical margin or two-step surgery.^20^ Standard surgical excision was performed, subsequently, the area was covered with an artificial dermis until confirmation of negative surgical margins. If tumors exhibit an aggressive biological behavior, it is recommended that a simple suture or skin graft be used as a reconstructive method to detect local recurrence as early as possible. Local flaps were also considered for other histological patterns.

The present study has several limitations. First, the data were only included in a retrospective single-center study with a small sample size. Furthermore, all patients were Japanese. In other countries, it is possible to excise well-defined and well-pigmented primary BCCs with a 2-mm narrow margin. However, BCCs may have different appearances and clinical characteristics. Therefore, the results should be carefully noted, since they apply only to a limited number of Japanese studies. Second, this study relied on information about permanent specimens cut by serial transverse sections (“bread-loafing”), and not all cross-sections could be evaluated (e.g., cases in which MMS was performed). Third, because the follow-up duration was short, the development of final local recurrence rate may change over time.

## Conclusions

Despite these limitations, the results of this study suggest that a 2-mm surgical margin is acceptable in cases of well-pigmented and well-defined primary BCC. It is important to note that well-pigmented and well-demarcated boundary BCCs, mostly nodular type in Japanese, can be treated in a single-step manner while preserving the maximal function and achieving cosmesis because this type has very clear margins and it rarely extends subcutaneous tissue. Considering the tumor characteristics, pigmentation, and border situation, combined with the presurgical examinations, a more reliable excision with a narrow surgical margin could be achieved. Recurrent lesions should be treated with caution to confirm margin negativity, and the most appropriate reconstructive methods should be carefully determined for each individual case.
